# Aurora-A signaling is activated in advanced stage of squamous cell carcinoma of head and neck cancer and requires osteopontin to stimulate invasive behavior

**DOI:** 10.18632/oncotarget.1896

**Published:** 2014-04-11

**Authors:** Chih-Yen Chien, Hsin-Ting Tsai, Li-Jen Su, Hui-Ching Chuang, Li-Yen Shiu, Chao-Cheng Huang, Fu-Min Fang, Chun-Chieh Yu, Huei-Ting Su, Chang-Han Chen

**Affiliations:** ^1^ Department of Otolaryngology, Kaohsiung Chang Gung Memorial Hospital, and Chang Gung University College of Medicine, Kaohsiung, Taiwan; ^2^ Kaohsiung Chang Gung Head and Neck Oncology Group, Kaohsiung Chang Gung Memorial Hospital, Kaohsiung Taiwan; ^3^ Graduate Institute of Systems Biology and Bioinformatics, National Central University, Jhongli, Taiwan; ^4^ Department of Medical Research, Cell Therapy and Research Center, E-Da Hospital, I-shou University, Kaohsiung, Taiwan; ^5^ Department of Pathology, Kaohsiung Chang Gung Memorial Hospital, and Chang Gung University College of Medicine, Kaohsiung Taiwan; ^6^ Department of Radiation Oncology, Kaohsiung Chang Gung Memorial Hospital, and Chang Gung University College of Medicine, Kaohsiung Taiwan; ^7^ Center for Translational Research in Biomedical Sciences, Kaohsiung Chang Gung Memorial Hospital, Kaohsiung Taiwan

**Keywords:** HNSCC, Aurora-A, osteopontin, CD44, ERK

## Abstract

The clinical significances, cellular effects, and molecular mechanisms by which Aurora-A mediate its invasive effects in HNSCC are still unclear. Here, we found that Aurora-A expression is significantly higher in tumor tissues on 14-microarray of HNSCC in Oncomine-databases. The activity of Aurora-A was not only found in HNSCC specimens, but also significantly correlated with advanced-T-classification, positive-N-classification, TNM-stage and the poor 5-year survival rate. HNSCC-microarray profile showed that osteopontin and Aurora-A exhibited positive correlation. Stimulation of HNC cells with osteopontin results in an increase in Aurora-A expression where localized at the centrosome. Functionally, Aurora-A had the abilities to stimulate cell motility in HNC cells through increase ERK1/2 activity under osteopontin stimulation. Conversely, depletion of Aurora-A expression by siRNAs suppressed ERK1/2 activity as well as inhibition of cell invasiveness. Treatment with anti-CD44 antibodies in HNC cells not only caused a decrease of mRNA/protein of Aurora-A and ERK1/2 activity upon osteopontin stimulation, but also affected the abilities of Aurora-A-elicited cell motility. Finally, immunohistochemical/Western-blotting analysis of human aggressive HNSCC specimens showed a significant positively correlation between osteopontin-Aurora-A and ERK1/2. These findings suggest that Aurora-A is not only an important prognostic factor but also a new therapeutic target in the osteopontin/CD44/ERK pathway for HNSCC treatment.

## INTRODUCTION

Head and neck squamous cell carcinoma (HNSCC) is common malignancy, and is cited as being the sixth most ordinary cancer worldwide. At present, the most important risk factors for the development of HNSCC remain tobacco use and alcohol consumption. Despite aggressive multidisciplinary treatment approaches, such as surgery, chemotherapy and radiotherapy, the 5-year overall survival remains at 50% over the past 30 years. Although HNSCC has a high incidence and mortality, the molecular mechanisms of this disease remain poorly understood. To improve the outcomes of patient, the identification of the molecular and genetic events involved in each step of HNSCC progression may help to understand the formation of HNSCC, and to develop the diagnostic markers and novel treatment strategies in the future.

Aurora-A also known as STK15 or STK6, located on 20q13, is a member of the serine/threonine kinase family. Aurora-A could regulate chromosome segregation and centrosomal spindle formation during mitosis [1-4]. Recent studies have shown that overexpression of Aurora-A in human epithelial cells results in centrosome number abnormality and aneuploidy production [5, 6]. Gene amplification or overexpression of Aurora-A often occur in many human cancers, including head and neck cancer[5-8]. Exogenous Aurora-A overexpression in mouse and rat fibroblast cells causes centrosome amplification and transformation *in vitro* as well as tumorigenesis *in vivo*[5, 6, 9]. Several studies also demonstrated that there is a positively correlation between Aurora-A expression and clinical aggressiveness, such as poorly differentiated tumor grade, and invasion in several cancers [5, 10-13],; however, some studies showed no correlation or an inverse correlation[10, 14-16]. These results suggested that the expression profile of Aurora-A and clinical significant in malignant cancers remains controversial. Furthermore, the activity of Aurora-A in human cancer tissues is still unclear. Recently, several reports have shown that Aurora-A associated with cellular proteins, such as p53, NF-kB, and PLK1 not only alters the physiologic functions of human cells, but also promotes tumor occurrence and malignant development[17-19]. However, the exact molecular mechanism underlying the induction of invasion by the Aurora-A in head and neck cancer cells; however has not been defined yet.

Osteopontin (OPN) or SPP1, a member of the small integrin binding ligand N-linked glycoprotein (SIBLING) family, contains aspartate and sialic-acid residues and contains unique functional domains[20]. OPN was originally characterized as an extra cellular matrix (ECM) protein secreted by transformed epithelial cells[21]. Highly expression of OPN has been detected in human cancer tissues and its expression correlates with advanced stage and poor outcome in thyroid, breast, prostate, lung, gastic, liver, oral, NPC, and colon cancer[22-26]. Histologic analysis of a variety of tumor tissues have shown that OPN expression is associated with invasion and metastasis in human cancers such as breast, stomach, lung, prostate, liver, and colon[20]. In addition, OPN has identified as a target for use as a serum biomarker in predicting cancer invasion. Gain and loss-of function experiments demonstrate that OPN mediates the metastatic spread of cancer cells. But in the context of HNSCC, the evidence has been less definitive. Furthermore, the signaling pathways by which OPN modulation to promote HNSCC metastasis and the relationship between its expression and other metastasis regulators are incompletely understood.

The aim of the present study was to examine the expression and activity of Aurora-A in a large cohort of HNSCC tissues and determine its relationships with clinicopathological variables, and patient's survival. Moreover, we also uncovered the novel biological functions of Aurora-A in head and neck cancer cells. These findings provide new evidence that Aurora-A overexpression contributes to the aggressiveness and development of HNSCC.

## RESULTS

### Increased expression of Aurora-A and its activity positively correlated with high-stage malignant HNSCC

**Figure 1:**
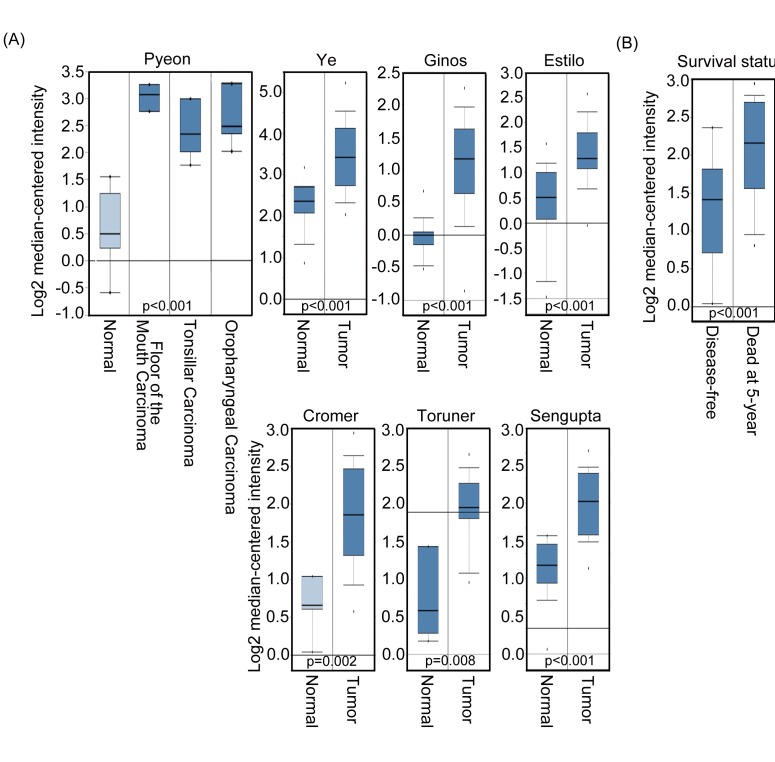
Aurora-A expression level is related to head and neck cancer (A) Selected datasets from the Oncomine cancer microarray database were mined to determine the alternations of Aurora-A in mRNA expression levels. Aurora-A transcript level was higher in tumor tissues than in normal tissues based on studies reported by Pyeon et al., (*p*<0.001), Ye et al., (*p*<0.001), Gino et al., (*p*<0.001), Estilo et al., (*p*<0.001), Cromer et al., (*p*=0.002), Toruner et al., (*p*=0.008), and Sengupta et al., (*p*<0.001). (B) The Aurora-A transcript level is low in 5 year disease-free patients with head and neck cancer.

To explore the contribution of Aurora-A to human squamous cell carcinoma of head and neck cancer, we first performed data mining and analyzed Aurora-A expression by using several published databases from the publicly available Oncomine database. [31] We used the Oncomine database to interrogate 14 human squamous cell carcinomas of head and neck cancer datasets for Aurora-A mRNA expression. We required a p-value of below 0.05 and a fold-change of 2 for Aurora-A gene expression compared to the control. The results indicated that a significant increase in Aurora-A mRNA level was observed in 7 out of 14 databases of head and neck samples compared to adjacent non-tumor (or normal) tissues (Figure 1A). In addition, Aurora-A expression level was higher in tumor tissues had poor survival compared with patients that remained disease-free (Figure 1B). Taken together, these data indicated that overexpressed Aurora-A participates in carcinogenesis of head and neck cancer and suggests that the high Aurora-A expression may indicate a poor prognosis.

**Figure 2:**
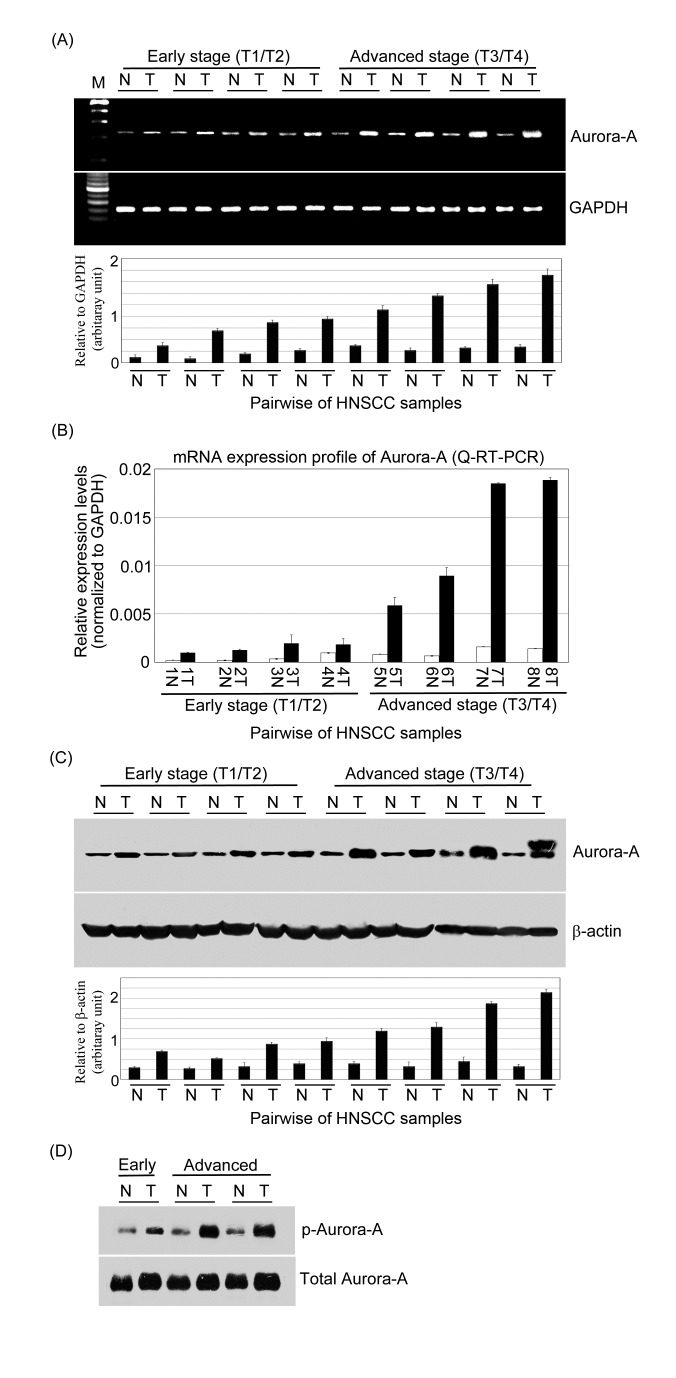
The expression levels of mRNA and protein and activity of Aurora-A are increased in advanced stage of HNSCC clinical samples (A) Semi-quantitative RT-PCR and (B) Q-RT-PCR analyzed the expressions of *Aurora-A* in HNSCC samples (T) versus that in adjacent non-cancerous tissue (N). *Aurora-A* overexpression was observed in 8-paired HNSCC samples. *GAPDH* was used as an internal loading control to normalize the amount of mRNA. Western blotting analysis of Aurora-A (C) and phosphor-Aurora-A (D) expressions in paired HNSCC patients. Total proteins were extracted from adjacent non-cancerous and tumor tissues and probed with polyclonal antibodies against Aurora-A and phosphor-Aurora-A. β-actin was used as a control. Relative quantities of Aurora-A mRNA and protein expression levels were represented HNSCC tissues and non-cancerous tissues.

To further evaluate the potential role of Aurora-A in HNSCC progression, first, we analyzed the expression of *Aurora-A* by semi-quantitative RT-PCR and real-time RT-PCR in 8-paired HNSCC specimens with early and advanced stages. Overexpression of Aurora-A mRNA was found in 8 of 8 cases (100%) of HNSCC tumor tissues compared with paired adjacent non-tumor tissues (Figure 2A and B). By Western blotting, Aurora-A protein was also observed upregulated in 8 of 8 HNSCC compared with their adjacent non tumor counterparts (Figure 2C). Furthermore, elevated Aurora-A mRNA and protein expressions are associated with advanced tumor stage versus early tumor stage (Figure 2A, B and C). We next determined the Aurora-A activity in paired- HNSCC tissues. The cell lysates from three-paired HNSCC tissues were prepared and active Aurora-A was determined from each sample with equal amounts of protein. As shown in figure 2D, Aurora-A activity was higher in tumor tissues of advanced stage than that in early stage. This result suggested that higher Aurora-A expression level was coincident with increased Aurora-A activity in tumor tissues.

**Figure 3:**
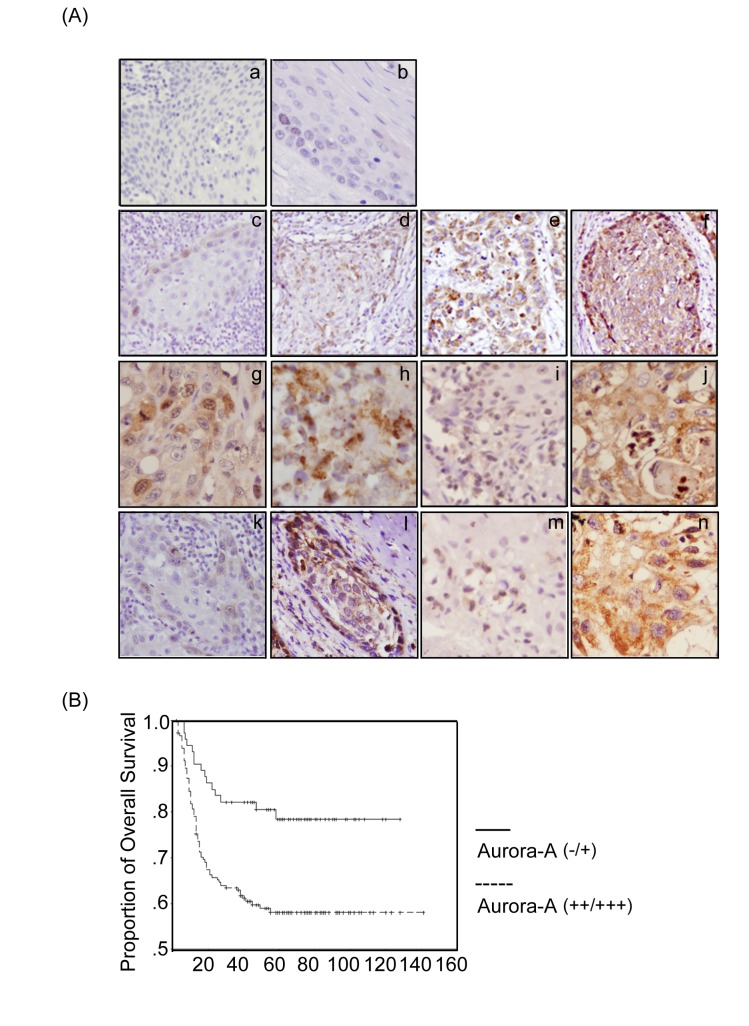
The expression of Aurora-A and its kinase activity are associated with poor prognosis in HNSCC patients by immunohistochemical staining (A) The tumor tissues of HNSCC and adjacent non-tumor tissues were collected and subjected to immunohistochemical staining with antibody against Aurora-A. Normal oral mucosa tissue (a) and adjacent non-cancerous tissue (b) were detected very weak Aurora-A expression in the cytoplasm. (c-f) Tumor tissues of HNSCC detected Aurora-A which had significant expression in the cytoplasm in the stage I, II, III, and IV, or in the nucleus (g) or with a punctuate staining in the cytoplasm (h). Aurora-A expression level was investigated in tumor tissues with lymph node-negative (i) or lymph node-positive (j). The expression profile of phosphor-Aurora-A in early stage (k), advanced stage (l), lymph node-negative (m), and lymph node-positive (n) were also examined. (Original magnification, 100X) (B) The overall survival was stratified in Aurora-A expression. The survival curve of HNSCC patients with strong expression (++/+++) of Aurora-A (dashed line) in tumor tissues was significantly shorter than that those patients with absent or weak (-/+) Aurora-A expression (solid line). There was a significant difference in the overall survival rate between the two groups (*p*<0.001) according to log-rank test.

Aurora-A overexpression was also confirmed by immunohistochemical staining of HNSCC tumors and adjacent non-tumor tissues. Two hundred and fifty-six HNSCC samples were analyzed. Representative results of Aurora-A immunostaining of HNSCC are shown in figure 3A. First, normal oral mucosa and the adjacent non-tumor tissues showed weak immunoreactivity for Aurora-A (Figure 3A, a and b).

Second, prominent staining was observed in the tumor samples (Figure 3A, c-h) compared to that in the adjacent non-cancerous tissues. Third, in the tumor samples, the protein expression of Aurora-A was positively correlation with tumor stage and node stage of the tumor cells (Figure 3A, c-f and i-j). Interestingly, it is found that Aurora-A was largely localized in cytoplasm of both tumor samples (Figure 3A, c-f) and the adjacent non-cancerous tissues (Figure 3A, b). Notably, in some cases, however, the tumor tissues showed that Aurora-A was expressed focally in the nucleus (Figure 3A, g). Moreover, a few tumor tissues, Aurora-A expression was also observed with punctate staining in the cytoplasm (Figure 3A, h). Similar results were also observed by using another Aurora-A antibody which produced by Abnova. To further confirm whether Aurora-A kinase activity was correlated with tumor stage in HNSCC, the immunohistochemical staining was also performed by using phosphor-Aurora-A antibody. It is positive correlation between Aurora-A kinase activity and advanced stage tissues of tumor (Figure 3A, k and l) and lymph node (Figure 3A, m and n) in HNSCC.

### Association of Aurora-A expression with clinicopathologic characteristics

Next, we classified the patients into two groups based on the immunohistochemical analysis: negative or low (–/+) Aurora-A expression and high (++/+++) Aurora-A expression to examine whether the expression of Aurora-A was associated with various prognostic factors. Patients with T3/T4 tumors, TNM stages III/IV, and lymph node positive (N+) had significantly higher expression of Aurora-A, as compared with patients with stage T1/T2 tumors (*p*<0.001), TNM stages I/II (*p*<0.001) and lymph node negative (N-) (*p*<0.001) (Table 1). Univariate analysis showed that advanced T classification (*p*<0.001), positive N classification (*p*<0.001), advanced TNM stage (*p*<0.001), and higher Aurora-A expression (*p*<0.001) indicated a significantly worse prognosis for 5-year overall survival of HNSCC patients (Table 2). In a cohort of 256 HNSCC specimens, the Aurora-A low-expression subgroup had a significantly better prognosis that Aurora-A high-expression subgroup by Kaplan-Meier analysis (Figure 3B). According to the Cox proportional hazards regression analysis, T stage (*p*=0.009), N stage (*p*<0.001) and Aurora-A (*p*=0.046) expressions were the independently associated with 5-year overall survival (*p*<0.001) (Table 3). These results clearly indicated that Aurora-A plays a role in the progression of this disease and the acquisition of an aggressive phenotype.

**Table 1:** Clinical profile and correlation between the clinicopathological features and expression of Aurora-A

Variables	No. of patients	Aurora-A Weak (-/+)	staining Strong (++/+++)	p value
Age (y) ≦59 ≧ 60	202 54	53 15	149 39	0.257
Gender Male Female	239 17	59 9	180 8	0.02
Tumor stage T1,T2 T3,T4	93 163	53 21	40 142	<0.001*
Nodal stage N(-) N(+)	153 103	68 5	85 98	<0.001*
TNM stage I,II III,IV	66 190	49 24	17 166	<0.001*
Histologic grade Well differentiated Moderately and Poorly differentiated	121 135	39 29	82 106	0.251

* Significant; No.: Number

**Table 2:** Univariate analysis of prognostic factors in HNSCC

Variables	No. of patients	Cumulative 5-year survival rate (%)	p value
Age (y) ≦ 59 ≧ 60	202 54	66.4 55.1	0.124
Gender Male Female	239 17	62.7 81.9	0.138
Tumor stage T1,T2 T3,T4	94 162	84.0 56.5	<0.001*
Nodal stage N(-) N(+)	153 103	77.1 44.7	<0.001*
TNM stage I,II III,IV	66 190	92.4 53.9	<0.001*
Histologic grade Well differentiated Moderately and Poorly differentiated	121 135	72.0 83.0	0.316
Aurora-A expression Weak Strong	68 188	92.6 53.3	0.0018*

* Significant; No.: Number

**Table 3:** Risk factors affecting 5-year overall survival rate determined by Cox's regression analysis

Variable	Relative Risk	95% Confident Interval	P value
T classification T3, T4 vs T1, T2	2.262	1.288-5.162	0.009*
N classification Positive vs Negative	2.851	1.968-5.228	< 0.001*
Aurora-A expression High vs Low	2.258	1.5-6.839	0.046*

* Statistically significant (p<0.05).

### Aurora-A promotes cell migration and invasive potential

**Figure 4:**
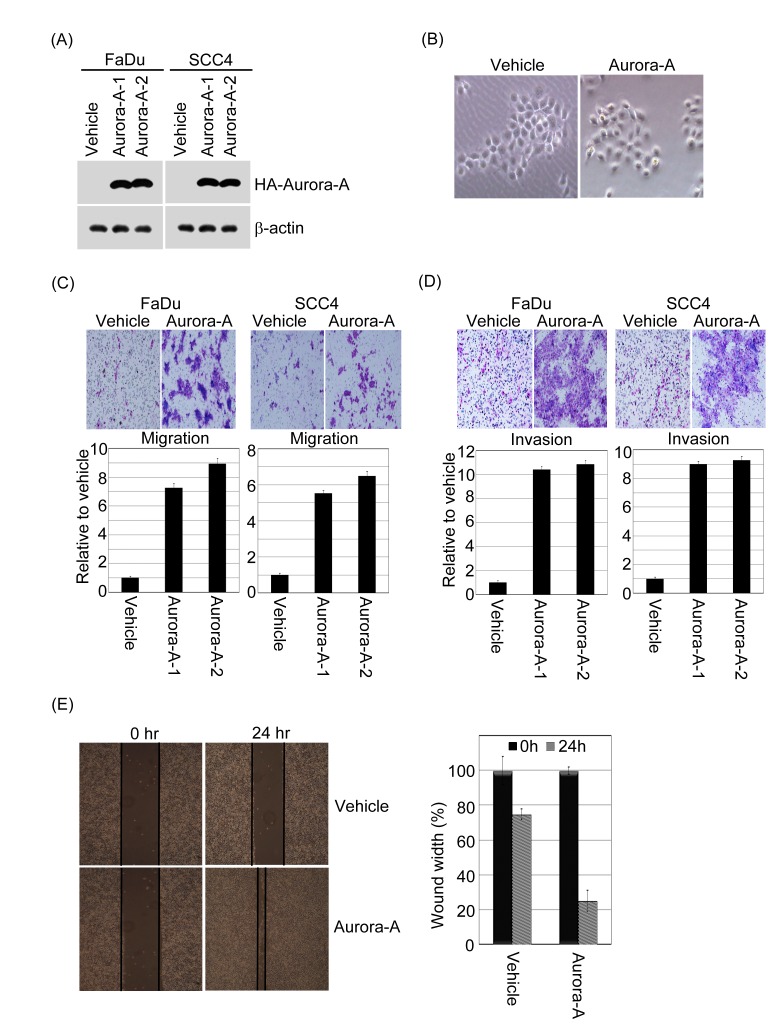
Exogenous Aurora-A regulates head and neck cancer cell migration and invasion (A) HA-tagged Aurora-A stable clone of FaDu and SCC4 cells were established. The cell lysates were subjected to immunoblot analysis with anti-HA antibody. β-actin is as an internal control. Representative images are shown from three independent experiments. (B) Phase-contrast images of monolayer cultures of FaDu cells expressing Aurora-A and vehicle control were shown. (C and D) For the migration assays, cells (FaDu-/SCC4-vehicle, FaDu-/SCC4-Aurora-A stable clones) were seeded into the top of a Transwell insert. For the invasion assays, cells were seeded after the addition of Matrigel. After 24 hour, the cells on top were scraped, and the cells that had migrated to the bottom were fixed and stained with Giemsa. The relative-fold migration and invasion values for the clones were normalized against the vehicle control and are represented diagrammatically. The migration and invasion photography results of FaDu-vehicle and FaDu-Aurora-A stable cells were shown (200x). (E) The result of wound-healing assay showed that migration ability of FaDu cells were promoted by Aurora-A overexpression at 24-hour time point. Representative images captured with 10X objective. All experiments were repeated at least thrice. The percentage of wound closure corresponds to the distance between wound edges in at least three randomly chosen regions relative to the distance at time 0 hour for each cell.

To evaluate the functional significance of Aurora-A in tumorigenesis, the gain-of function of Aurora-A in FaDu and SCC4 cell lines were established (Figure 4A). Interestingly, Aurora-A-overexpression FaDu cell was displayed with strikingly altered cell morphology. Steady-expressed Aurora-A cells were decreased in cell size and appeared to be more spindle-shaped and had increased intercellular separation compared to vehicle control clones, which were round-shaped (Figure 4B). A similar pattern of morphological changes was also observed in Aurora-A-SCC4 transfectants (data not shown). The Aurora-A transfectants exhibited a stretched spindle-like morphology, suggesting that Aurora-A expressing may cause the motility of cancer cells.

**Figure 5:**
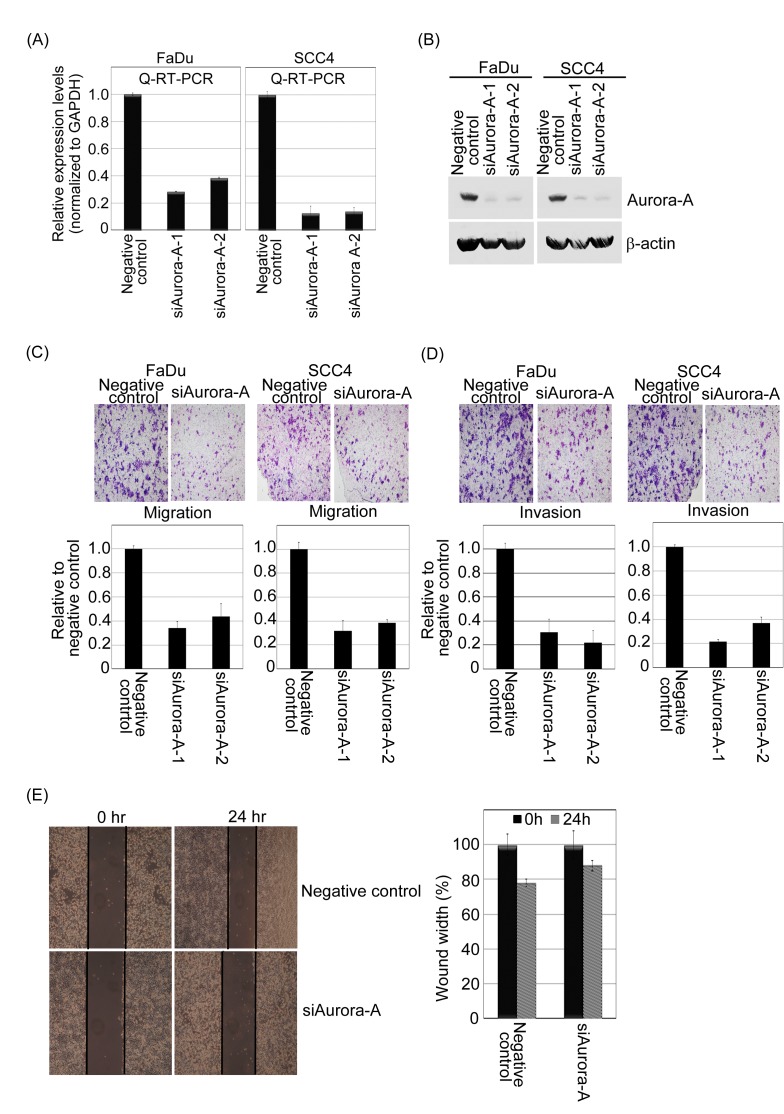
The knockdown of endogenous Aurora-A inhibits migration and invasion of head and neck cancer cell lines (A and B) A negative control siRNA plus two *Aurora-A* siRNAs were transfected into FaDu and SCC4 cells for 24 hour. After transfection, endogenous mRNA of Aurora-A was detected by Q-RT-PCR, and Western blotting approach using Taq-Man Aurora-A probe, anti-Aurora-A and β-actin antibodies. (C and D) The relative-fold migration and invasion of FaDu-/SCC4-siAurora-A was normalized against the values for the negative control cells and are represented diagrammatically. All of the data represent the mean ± s.d. of three independent experiments. The migration and invasion photography results of negative control and siAurora-A-FaDu stable cells were shown (200x). (E) Wound healing assays of the FaDu cells transfected with negative control or siAurora-A. Representative images captured with 10X objective at the time of wounding or 24 hour after. All experiments were repeated at least thrice. The percentage of wound closure corresponds to the distance between wound edges in at least three randomly chosen regions relative to the distance at time 0 hour for each cell.

Next, we analyzed whether increased Aurora-A expression affects metastatic parameters, including migration and invasion. First, to test whether enhanced Aurora-A increases cell migration and invasive abilities, the Transwell coating with or without Matrigel were applied. The representative fields of cell migration and invasion experiments are shown (Figure 4 C and D). Overexpression of Aurora-A-FaDu and Aurora-A-SCC4 cells led to a significant increase in head and neck cancer cell migration and invasion (Figure 4C and D). Quantitatively speaking, the Aurora-A-FaDu and Aurora-A-SCC4 transfectants induced cell migration at a rate that were about 7.5-9.5-fold and 5.5-6.5-fold higher than in the vehicle cells (Figure 4C); moreover, the invasive ability of the Aurora-A-FaDu and Aurora-A-SCC4 transfectants were approximately 10.5-11-fold and 9-9.5-fold higher than that of the vehicle controls (Figure 4D). In addition, the migratory ability of Aurora-A-FaDu cells was analyzed in wound healing assays. At 24 hour post-wounding, FaDu-Aurora-A cells achieved near-complete wound closure, whereas vehicle control cells did not (Figure 4E). Similar results were also observed in SCC4 cells (data not shown). To inquire whether abolition of Aurora-A has an opposite effect on tumorigenesis, we used siRNA approach to inhibit endogenous Aurora-A expression and assayed the migratory and invasive abilities of FaDu and SCC4 cells. The endogenous mRNA and protein expression levels of Aurora-A in FaDu and SCC4 cells transfected with siRNAs targeting Aurora-A were significantly reduced (Figure 5A and B). Knockdown of endogenous Aurora-A in FaDu and SCC4 cells led to a significant diminished in wound healing, migration and invasion (Figure 5C, D and E). Taken together, these results strongly suggest that Aurora-A plays an important role in human head and neck cancer cell migration and invasion.

### By using the concept of syn-expression to explore osteopontin as the upstream regulator of Aurora-A

**Figure 6:**
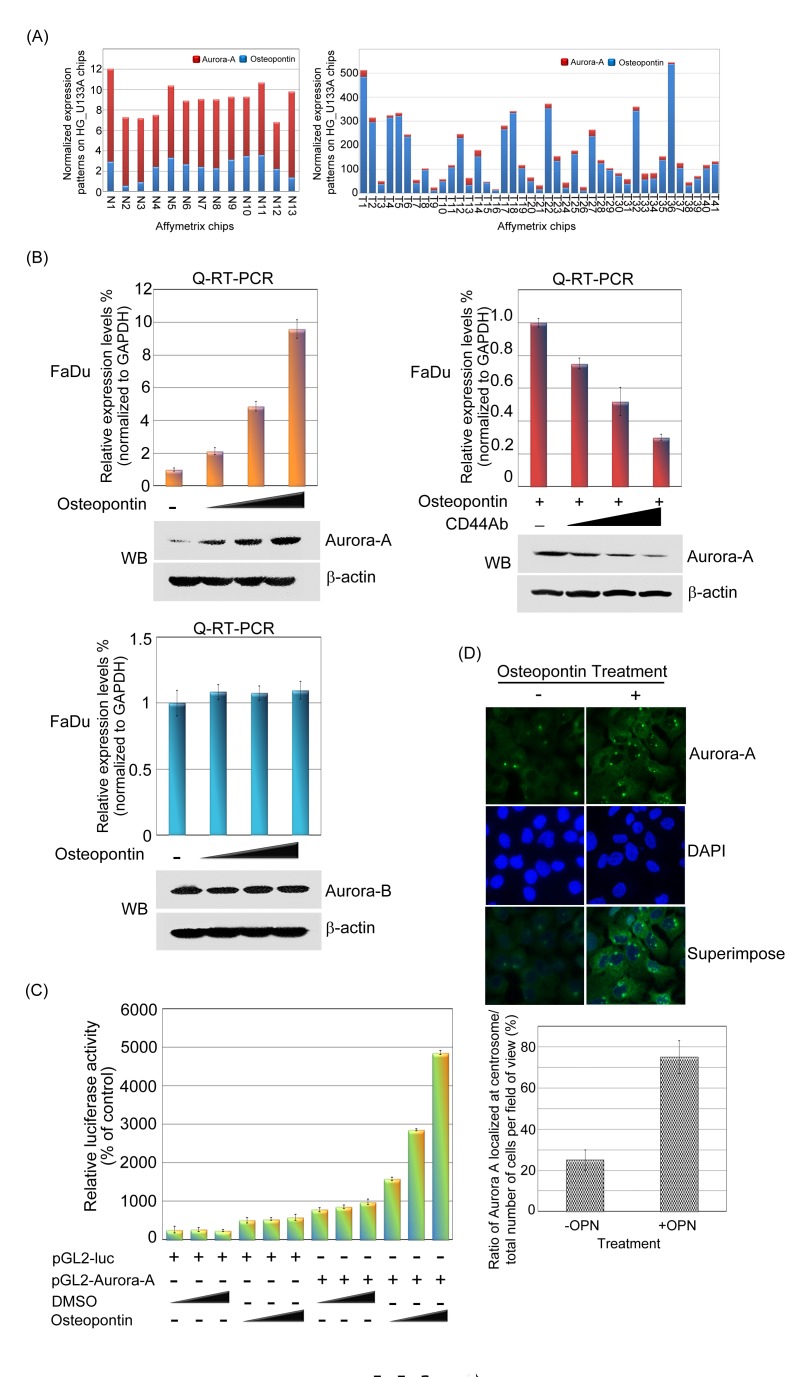
Aurora-A is up-regulated by osteopontin stimulation in human head and neck cancer cells (A) The microarray expression patterns of Aurora-A and osteopontin in head and neck cancer were shown. The results were normalized against the expression patterns of 54 chips (HG_U133A). N: normal tissues. T: tumor tissues. (B, left panel) The mRNA and protein expression levels of Aurora-A and Aurora B were examined by Q-RT-PCR and Western blotting in FaDu cells in osteopontin dose-dependent manner. The results were normalized against the expression level of *GAPDH* mRNA in each osteopontin-treated cell. Using the same panel, the total proteins were extracted from FaDu cells and probed with antibodies against Aurora-A, Aurora B and β-actin. β-actin was used as a control. Data are representative of three independent experiments done in triplicate. (B, right panel) Serum-starved FaDu cells were pre-treated with or without various concentrations of CD44 antibodies for 2 hour, then cells stimulated with 20 ng/ml osteopontin for 15 min. Cells were harvested and performed Western blotting. (C) Luciferase assays were done to detect promoter activity of Aurora-A in transfected FaDu cells in the presence or absence of osteopontin. The luciferase activity in 1μg of cell lysate was normalized to β-galactosidase activity. Data are representative of three independent experiments done in triplicate. (D) Immunofluorescence imaging of Aurora-A and nuclei of FaDu cells with or without osteopontin stimulation. Ratio of Aurora-A localized at centrosome was quantified from 20 images per condition.

To identify perturbed pathways, differential gene-gene co-expression has been implemented for studying changes between different diseases and biological conditions. [32] Here, we employed head and neck microarray database to gain insight into the functional concordance of co-expressed genes of Aurora-A. [33] We examined whether the mRNA expression profile of *Aurora-A* correlated with any ligand in the microarray database of head and neck cancer. Of these co-expressed genes, the top candidate was *osteopontin*, which was highly positively correlated with the mRNA expression level of *Aurora-A* in paired head and neck cancer patients (Figure 6A; and Supplementary Table 1) (*p*<0.001). This raises the possibility that these two molecules are functionally linked or in the same pathway.

First, we were interested in whether osteopontin could modulate Aurora-A gene and protein expressions in head and neck cancer cells. As shown in the figure 6B, the mRNA and protein expression levels of Aurora-A were increased in an osteopontin dose-dependent manner in FaDu cells by Q-RT-PCR and Western blotting (Figure 6B, upper left panel). Similar results were also obtained in the SCC4 cells (Supplementary Figure 1A).

We next asked whether CD44, a binding receptor of osteopontin could be contributed to the regulation of Aurora-A expression in head and neck cancer cells. The CD44 receptors were blocked using neutralizing antibodies, and analyzed Aurora-A mRNA and protein expressions in FaDu cells upon osteopontin stimulation. The results demonstrated that upregulation of Aurora-A in the presence of osteopontin was inhibited by the simultaneous addition of CD44 antibodies in a dose-dependent manner (Figure 6B, upper right panel). On the contrary the mRNA and protein level of Aurora B, an Aurora kinase family, was not influence by stimulating with osteopontin (Figure 6B, bottom panel and Supplementary Figure 1B). In fact, osteopontin does not have similar expression patterns with Aurora B, at least not in our analyzed microarray dataset.

Next, to investigate the role of osteopontin in regulating Aurora-A transcription, we determine whether the promoter activity Aurora-A could be regulated by osteopontin stimulation. We transfected the Aurora-A promoter-luciferase constructs into FaDu cells following osteopontin stimulation in a dose-dependent manner. The data showed that a significant activation of the Aurora-A promoter was detected under osteopontin stimulation (Figure 6C). To confirm whether CD44 mediated the promoter activation of Aurora-A upon osteopontin stimulation, FaDu cells containing Aurora-A promoter with anti-CD44 blocking antibody or isotypes IgG antibody in the presence of osteopontin were monitored. Anti-CD44 antibodies resulted in a significant decreased of Aurora-A promoter activity in the presence of osteopontin stimulation, compared to isotype IgG antibody group (Supplementary Figure 2). These results illustrated that Aurora-A is one of the downstream targets of the osteopontin signaling pathway in human head and neck cancer cells.

### Aurora-A centrosome accumulation is required for osteopontin-stimulation in head and neck cancer cell

Aurora-A is often considered to be redundant in function depend on its cellular localization [34-37]. To further characterize the expression of the Aurora-A in human head and neck cancer cell under osteopontin stimulation, we investigated its subcellular localization by means of indirect immunofluorescence microscopy. Here we observed that cells without osteopontin stimulation, the endogenous Aurora-A exhibited a primarily cytosolic localization with some centrosome staining. Conversely, in the present of osteopontin treatment, Aurora-A was induced with dramatic increasing in centrosome staining (Figure 6D). Such a pattern was similar to that observed in SCC4 cells (Supplementary Figure 3). Furthermore, the ratio of Aurora-A-positive cells which localized in centrosome over the total number of cells was significantly increased in treated conditions compared with control group which without osteopontin treatment (*p*<0.001) (Figure 6D). These observations prompted us to discern the role of Aurora-A in osteopontin-inducing head and neck cancer cells.

### Osteopontin-elicited Aurora-A overexpression enhances head and neck cancer cell migration and invasion

**Figure 7:**
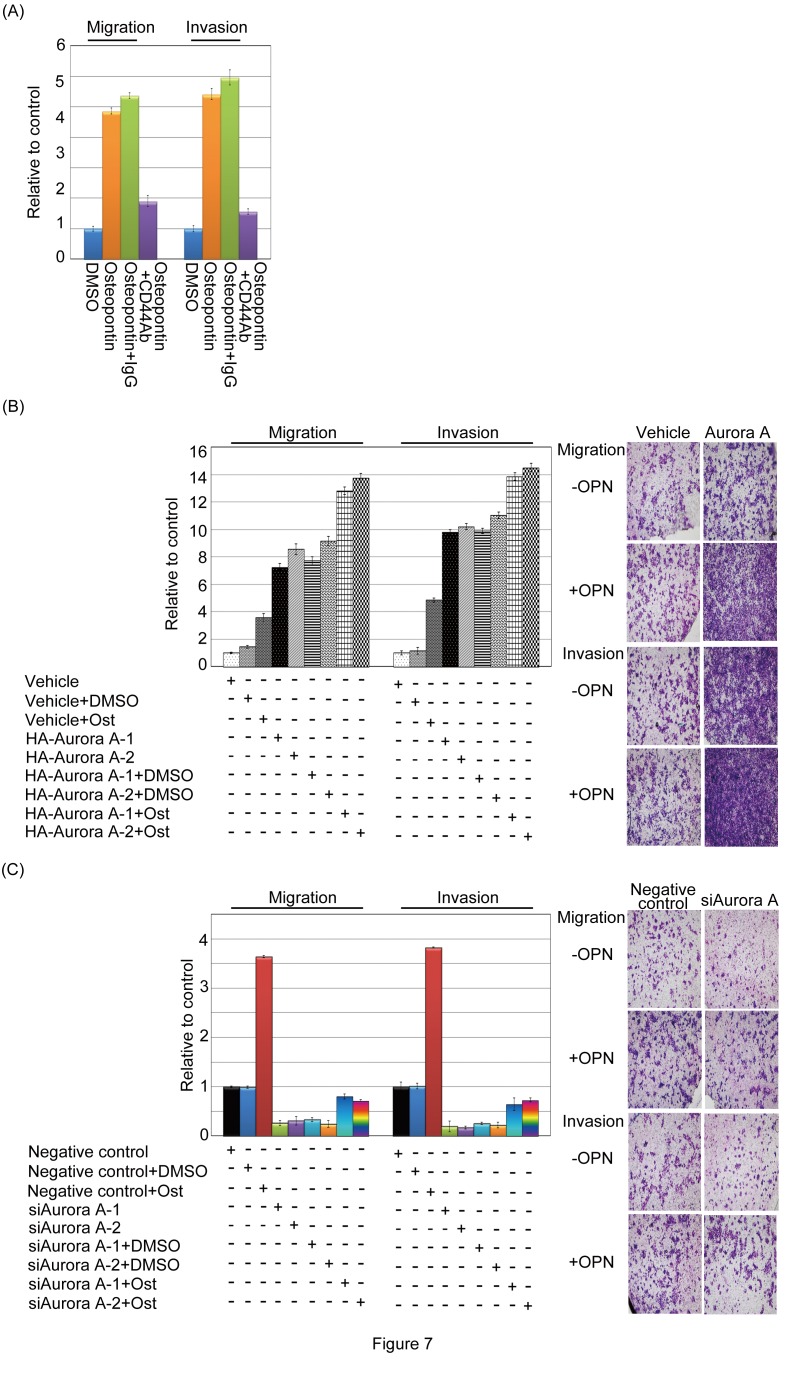
Osteopontin enhanced Aurora-A-induced migration and invasion in head and neck cancer cells (A) Serum-starved FaDu cells were pre-treated with or without CD44 antibody for 2 hour, then cells stimulated with 20 ng/ml osteopontin for 3 hour. The migration and invasion relative-folds were normalized against vehicle cells. (B and C) For the migration assays, 5 x 10^3^ cells (Vehicle-FaDu, Aurora-A-FaDu, negative control-FaDu, and siAurora-A-FaDu) with or without osteopontin stimulation were seeded into the top of a Transwell insert. After 24 hours, the cells on top were scraped, and the cells that had migrated to the bottom were fixed and stained with Giemsa. For the invasion assays, 1 x 10^4^ cells were seeded after the addition of Matrigel. The relative-fold migration/invasion values for the stable clones were normalized against the vehicle/negative control cells and are represented diagrammatically. The migration and invasion photography results were shown (200x). All of the data represent the mean ± s.d. of three independent experiments. Ost: Osteopontin.

As we known, osteopontin participates in angiogenic process in human cancer cells, such as migration and invasion. To explore the biological importance of osteopontin-induced upregulation of Aurora-A, we investigated the effects of Aurora-A on motility in the presence or absence of osteopontin. First, we tested whether parental FaDu cells could exhibit cell motility after osteopontin stimulation. In figure 7A, the FaDu parental cells displayed more migration and invasive abilities in the presence of osteopontin than DMSO or osteopontin combined with isotype IgG and CD44 Ab, suggesting that FaDu cell stimulated with osteopontin could enhance cell motility, which was paralleled by an increase in Aurora-A levels. Furthermore, overexpressed Aurora-A stable cells in the absence or presence of osteopontin stimulation were analyzed the cell motility in a Transwell chamber. The results demonstrated that Aurora-A stable cells with osteopontin stimulation had a higher migration and invasion abilities than that the Aurora-A alone or vehicle control (Figure 7B). However, compared with siAurora-A transfectants, the migratory and invasive abilities of siAurora-A cells were slightly increased after cell-treated with osteopontin (Figure 7C). Taken together, these results strongly suggest that Aurora-A is required for proper osteopontin-dependent signaling, and that it contributes to cell migration and invasion in head and neck cancer cells.

### The activation of ERK1/2 modulated by Aurora-A participates in head and neck cancer cell migration and invasion

**Figure 8:**
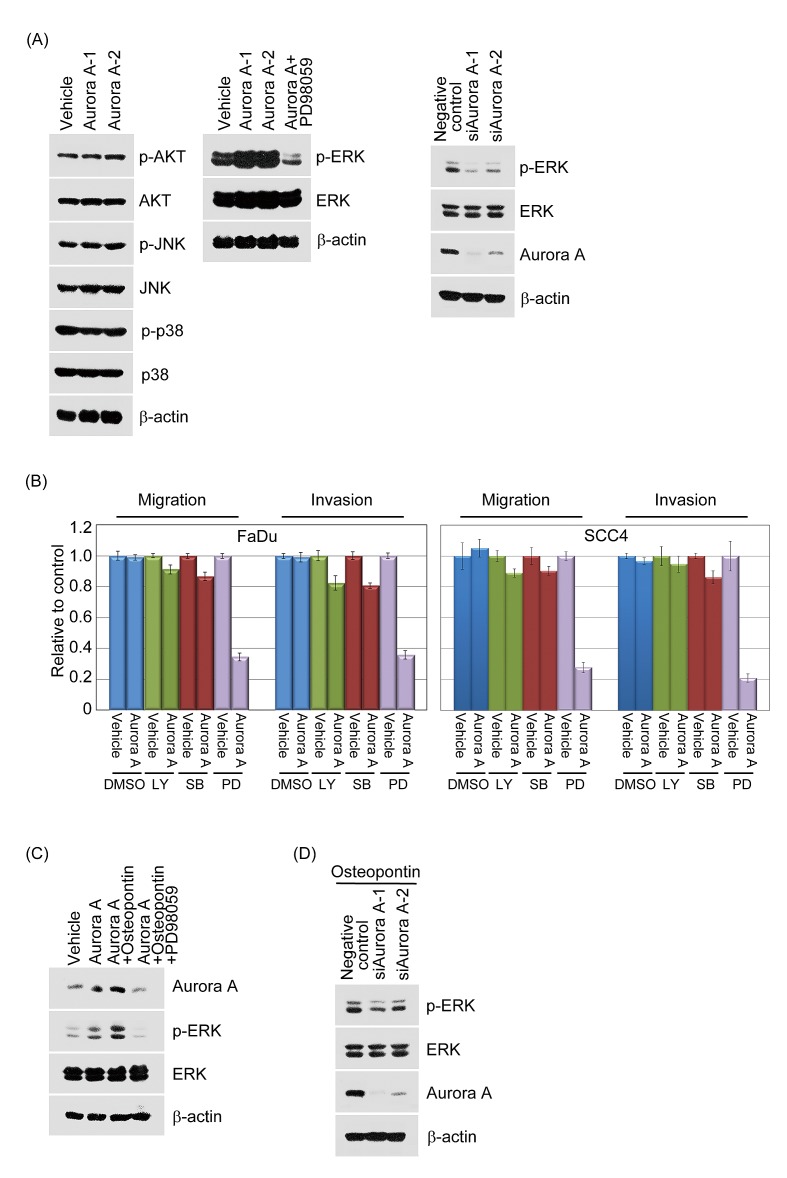
Aurora-A modulates cell migration and invasion through enhanced ERK activity (A, left panel) Vehicle-FaDu or Aurora-A-FaDu cells were serum-starved for 24 hour and following the Western blotting analysis. The protein lysates were subjected to immunoblot analysis to detect the phosphorylated or unphosphorylated forms of AKT, JNK, and p38. (A, middle panel) Vehicle-FaDu or Aurora-A-FaDu cells were serum-starved for 24 hour and treated with or without the ERK inhibitor, PD98059 for 24 hour. The protein lysates were detected the unphosphorylated or phosphorylated form of ERK. β-actin was used as the internal loading control (A, right panel) Negative control-FaDu or siAurora-A-FaDu cells were serum-starved for 24 hour and following the Western blotting analysis. The protein lysates were detected the unphosphorylated or phosphorylated form of ERK. (B) Vehicle-FaDu, Vehicle-SCC4, Aurora-A-FaDu and Aurora-A-SCC4 transfectants were serum-starved and treated with the indicated inhibitors, SB202190, PD98059, and LY294002 or solvent for 24 hour. The migration and invasion ratios of vehicle-FaDu, Aurora-A-FaDu, vehicle-SCC4, and Aurora-A-SCC4 transfectants were determined as previously described. (C) Vehicle-FaDu and Aurora-A-FaDu transfectants were pre-treated with or without osteopontin. After 15 min, PD98059 was added and cells were further incubated for 2 hour. Total cell lysates were subjected to immunoblot analysis for Aurora-A, the unphosphorylated and phosphorylated forms of ERK. β-actin was used as the internal loading control. (D) A negative control plus two Aurora-A siRNAs were transfected into FaDu cells for 24 hours. After transfection, cells were treated with osteopontin for 15 min. Western blotting was performed as in (C).

To identify Aurora-A downstream targets through which Aurora-A executes its biological functions, a panel of phosphorylated antibodies, which delineate the activation state of the signaling pathways, was employed to screen the possible involvement of four known kinase signaling pathways, extracellular signal-regulated kinase (ERK), c-Jun NH2-terminal kinase (JNK), p38 and AKT. Vehicle and Aurora-A transfectants were cultured with serum starvation for 24 hour. The cell lysates were examined by Western blotting with unphosphorylated/phosphorylated antibodies of ERK1/2(p-202/204), JNK(p-183), p38(p-180) and AKT(p-473). The activities of AKT, JNK and p38 were not significantly distinct between Aurora-A-FaDu transfectants and vehicle. In contrast, ERK1/2 phosphorylation was raised in Aurora-A stable cells when compared with vehicle-FaDu control cells whereas no significant alteration in total ERK1/2 protein level was observed (Figure 8A).

To provide additional proof for the participation of ERK1/2 in Aurora-A-elicited cell metastasis, we took three approaches. First, we investigated whether the activation of ERK1/2 was mediated by Aurora-A. As shown in Figure 8A middle panel, Aurora-A-elicited ERK1/2 (Thr 202/Tyr 204) phosphorylation was partially abolished by PD98059, an ERK inhibitor. Second, we next determined whether endogenous Aurora-A executes a similar biological function. Ablation of endogenous Aurora-A by Aurora-A-mediated siRNAs resulted in a significant decrease in the activation of ERK1/2 in FaDu cells (Figure 8A, right panel). To examine whether Aurora-A-elevated ERK activation is involved in cellular motility, the abilities of migration and invasion of Aurora-A in FaDu cells was evaluated by Transwell chamber. Figure 8B illustrated that Aurora-A-FaDu transfectants had increased levels of cell migration and invasion, which were strongly inhibited by incubation with PD98059, and to a much lesser extent with SB202190 and LY294002. Similar results were also observed in SCC4-Aurora-A stable cells (Figure 8B, right panel).

Next, we took two approaches to provide additional evidence for the participation of ERK in Aurora-A-raised cell migration and invasion upon osteopontin stimulation. First, we examined whether increased ERK1/2 activity could be noted in Aurora-A-FaDu stable cells upon osteopontin treatment. The results indicated that enhanced ERK1/2 phosphorylation was observed in Aurora-A transfectants upon osteopontin stimulation, compared with the vehicle and Aurora-A treated transfectants (Figure 8C). However, Aurora-A-elicited ERK1/2 phosphorylation was completely abolished by PD98059 upon osteopontin stimulation (Figure 8C). Secondly, we investigated if Aurora-A siRNAs might affect endogenous ERK1/2 activation in FaDu cell, with or without osteopontin stimulation. Our data showed that the activity level of ERK in Aurora-A-depleted cells was slightly increased upon osteopontin stimulation compared with negative control (Figure 8D), probably due to low residual Aurora-A present in cells. Taken together, these results seem to indicate that ERK activation may participate in an osteopontin-modulated Aurora-A signaling pathway in head and neck cancer cells.

### The activity of ERK1/2 could be triggered by osteopontin and promotes head and neck cancer cells motility via CD44

**Figure 9:**
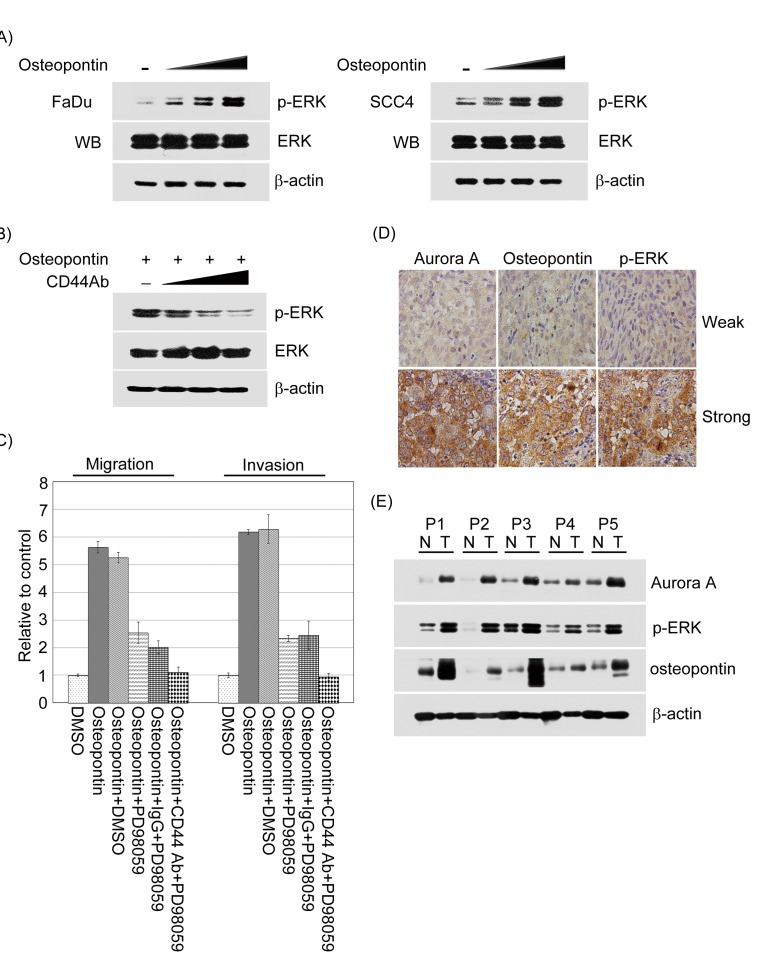
The activation of ERK regulated by osteopontin could promote cell motility in head and neck cancer cells and has a highly correlation with Aurora-A and osteopontin expressions in human aggressive HNSCC (A) The activity of ERK was examined by Western blotting in FaDu and SCC4 cells treated with or without osteopontin stimulation in dose-dependent manner. The total protein was extracted from FaDu and SCC4 cells and probed with antibodies against unphosphorylated or phosphorylated form of ERK. β-actin was used as a control. Data are representative of three independent experiments done in triplicate. (B) Serum-starved FaDu cells were pre-treated with or without various concentrations of CD44 antibodies for 2 hour, then cells stimulated with 20 ng/ml osteopontin for 15 min. Cells were harvested and performed Western blotting. (C) Serum-starved FaDu cells were pre-treated with isotype IgG or CD44 antibodies combining with PD98059 for 2 hour, then cells stimulated with 20 ng/ml osteopontin for 15 min. The migration and invasion relative-folds were normalized against vehicle cells. (D) Osteopontin, Aurora-A, and phosphorylated ERK expressions in human aggressiveness HNSCC specimens. Immunohistochemical staining using anti-osteopontin, anti-Aurora-A or anti-phosphorylated ERK antibodies was carried out with 50 HNSCC tissue sections. Photographs of weak and strong stainings for osteopontin, Aurora-A, and phosphorylated ERK in the sections are shown. (E) The osteopontin, Aurora-A, and phosphorylated ERK protein expression levels were determined by Western blot analysis from five frozen tissue samples collected from patients with aggressive HNSCC.

To further confirm if ERK activity might serve as a critical mediator of osteopontin-induced cell migration and invasion in head and neck cancer cells, the protein activity of ERK1/2 was measured by Western blotting using FaDu and SCC4 cells upon osteopontin stimulation. As shown in the Figure 9A, the protein activity of ERK1/2 was upregulated in an osteopontin dose-dependent manner in FaDu and SCC4 cells. We next questioned whether CD44 could be attributed to modulate the ERK1/2 activity induced by osteopontin in head and neck cancer cells. The results showed that the activity of ERK1/2 in the presence of osteopontin was suppressed by simultaneous addition of CD44 antibody in a dose-dependent manner (Figure 9B).

To confirm examined whether CD44 participates in mediating ERK function upon osteopontin stimulation, FaDu cells treated with anti-CD44 or isotypes IgG antibodies in the presence of osteopontin or PD98059 and monitored cell motility. Anti-CD44 antibodies resulted in a significant inhibition of head and neck cancer cell motility in the presence of osteopontin stimulation combining with PD98059, compared to parental cells treated with isotype IgG antibody (Figure 9C). Taken together, these results indicate that ERK activation for head and neck cancer cell migration and invasion is required for osteopontin/CD44-dependent signaling pathway.

### Concordant expression of high levels of osteopontin, Aurora-A, and active ERK in associated with aggressive HNSCC

To evaluate the potential relevance of the above findings in aggressive HNSCC, we analyzed the protein expressions of osteopontin, Aurora-A, and phosphorylated ERK1/2 in 50 samples from patients with aggressive HNSCC by immunohistochemical staining. A representative staining results of weak and strong are shown in Figure 9D. It is indicated that osteopontin, Aurora-A, and phosphorylated ERK1/2 were coexpressed in aggressive HNSCC specimens, suggesting that they are, in most cases, related events in the development of HNSCC. The correlation between each paired IHC scores of osteopontin, Aurora-A, and phosphorylated ERK were analyzed by Spearman's rank tests. The result showed that there were positive correlations between osteopontin and Aurora-A (rho=0.792, *p*<0.001), and phosphorylated ERK (rho=0.682, *p*<0.001) and Aurora-A and phosphorylated ERK (rho=0.834, *p*<0.001) (Table 4). Those patients with a high osteopontin score had a significantly higher Aurora-A and phosphorylated ERK score. Next, we performed Western blot analyses using total protein extracts from five tumor samples collected from patients with aggressive HNSCC. As shown in figure 9E, osteopontin expression was correlated with Aurora-A and phosphorylated ERK expression. Taken together, these results suggest that there is a significant positively correlation among osteopontin, Aurora-A and phosphorylated ERK in aggressive human HNSCC.

**Table 4:** The correlation between Aurora-A, Osteopontin, and phosphorylated-ERK

		Aurora-A	Osteopontin	p-ERK
Aurora-A	Spearman's rank correlation	1		
	Sig. (2-tailed)	.		
	Number	50		
Osteopontin	Spearman's rank correlation	0.792**	1	
	Sig. (2-tailed)	<0.0001	.	
	Number	50	50	
p-ERK	Spearman's rank correlation	0.834**	0.682**	1
	Sig. (2-tailed)	<0.0001	<0.0001	.
	Number	50	50	50

## DISCUSSION

Aurora-A has been reported to be overexpressed in a number of different types of human malignancies, such as, lung small cell lung cancer (NSCLC), esophageal squamous cell carcinoma, and gastric cancer, hepatocellular carcinoma, bladder cancer, glioma, pancreatic cancer, nasopharyngeal carcinoma, and head and neck cancer[5, 10, 11, 14, 15, 38-41], and has been illustrated to be a poor prognosis markers. However, the activity and role of Aurora-A in invasive behavior and its relationship with clinical significance in aggressive HNSCC has not yet been explored. In this present study, we demonstrated the systematic survey of Aurora-A expression and found that (i) The advanced tumor tissues of HNSCC patients had high expression and activity of Aurora-A and its distribution mainly was detected in the cytoplasm in tumor cells. (ii) Higher expression of Aurora-A in HNSCC patients had a significantly worse 5-year survival rate compared with HNSCC patients with low expression of Aurora-A both in univariate and in multivariate analysis (*p*=0.0018 and *p*=0.046, respectively). (iii) Aurora-A overexpression in head and neck cancer cells promotes cell migration and invasion. (iv) In contrast, inhibition of endogenous Aurora-A levels using siRNAs resulted in decrease in migratory and invasive abilities in head and neck cancer cells. (v) Aurora-A expression could be modulated by osteopontin-CD44 dependent pathway for enhancing cell motility and accumulating in the centrosome. (vi) The migratory and invasive abilities of Aurora-A-mediated head and neck cancer cells are dependent on the ERK1/2 pathway upon osteopontin stimulation. (vii) Aurora-A-induced cell metastasis is correlated with elevated osteopontin and phosphorylated ERK1/2 expressions in aggressive HNSCC specimens. Collectively, these results strongly suggested that activation of osteopontin-Aurora-A-ERK signaling confers growth and metastatic advantages to HNSCC.

Aurora-A is participated in centrosome functions, such as duplication, maturation, and bipolar spindle assembly, and chromosome segregation during normal cell mitosis[14] However, its abnormalities such as gene amplification and overexpression may result in progression of malignant tumors. There is a growing body to evidence that up-regulation of Aurora-A can lead to an invasive and metastatic phenotypes of human cancer cells including head and neck cancer and ESCC, and contribute to shorten disease-free survival in human cancer patients[35, 41]. Interestingly, the Aurora-A kinase polymorphism (Phe31/Ile) was significantly associated with tumor recurrence, and risk of death, and disease-free survival in ESCC [42] According to these reports, it is support our conclusion that abnormal Aurora-A expression promotes the development of tumor cells and clinical aggressiveness in HNSCC.

Using an immunochemical staining approach, we found that Aurora-A protein was strongly overexpressed in the HNSCC tumor tissues, compared to adjacent non-tumor tissues. These findings were further confirmed by semi-quantitative RT-PCR, Q-RT-PCR, and Western blotting analysis. Furthermore, overexpressed Aurora-A in tumor tissues of HNSCC was associated with the grades of tumor, lymph node, and patient survival, suggesting that Aurora-A may play a useful marker for assessing advanced stages of HNSCC and determining the prognosis of this disease. Our data showed that overexpressed Aurora-A protein in most HNSCC tumor tissues was primarily distributed in the cytoplasm compared to that found in nucleus of tumor (~10-15%) and the adjacent non-tumor tissues (~5-10%). Similar finding also observed in other human cancer tissues, such as bladder, colorectal and ESCC[5, 43, 44]. In contrast to these studies, several reports indicated that Aurora-A accumulated in the nucleus and associated with advanced cancer stage[1, 37]. These differences might result from a diversity of methodology and affinity of the primary antibodies.

In order to understand the connection between Aurora-A and HNSCC metastasis in more detail, it will be of great interest to identify signaling cascades by which Aurora-A regulates migration and invasion. The data, for the first time, illustrate that invasion by Aurora-mediated head and neck cancer cell is dependent on the ERK pathway. Conversely, cell invasion induced by ERK signaling was completely abrogated in Aurora-A-overexpressing head and neck cancer cells by PD98059. Lines of evidence have suggested that hyperactivation of ERK acted as a crucial factor in the process of invasion in human cancer tissues and cell lines[38, 45]. Recently, MAPK, such as ERK, was reported to regulated Aurora-A expression in pancreatic cancer cell and nasopharyngeal carcinoma cell[38, 46]. However, we did not observe that MAPK could modulate Aurora-A expression in our current study (data not shown). Here, we also found that the mRNA and protein expressions of Aurora-A and activity of ERK in head and neck cancer cells could be regulated by osteopontin stimulation in a dose-dependent manner. Furthermore, Aurora-A transfectants with osteopontin stimulation could enhance head and neck cancer cell migration and invasion compared to Aurora-A stable cells. Importantly, blockade of CD44 receptor caused significant inhibitions of Aurora-A-induced cell migration and invasion in the presence of osteopontin stimulation in head and neck cancer cells. In this regard, our results show that CD44 provides an important link for understanding the role of osteoponint/Aurora-A in cell metastasis. Finally, the Western blotting, and immunohistochemical analysis showed a significant correlation that the expression of osteopontin was positively correlation with the expression of Aurora-A and phosphorylated ERK in aggressive HNSCC specimens. These results strongly suggested that the activation of osteopontin-Aurora-A-ERK signaling is associated with progression, especially invasive capacity in HNSCC.

## CONCLUSION

In summary, our results demonstrated that strong expression and activity of Aurora-A were associated with advanced tumor stage, lymph node stage, and poor survival in HNSCC specimens. The abnormality of Aurora-A expression in head and neck cancer cell is able to promote cell migration and invasion. Most importantly, the quantity of Aurora-A in mammalian cells may serve as a crucial mediator of osteopontin/CD44-dependent biological events via the activation of ERK signaling pathway, resulting in the progression of HNSCC. These results suggested that Aurora-A is a predictor of HNSCC invasion and may be a potential therapeutic target for blocking HNSCC invasion.

## MATERIAL AND METHODS

### Patients and tumor samples

The study population included 256 patients who underwent primary surgical resection between October of 1996 and August of 2005 for the treatment of HNSCC without previous radiotherapy and/or chemotherapy. Clinicopathological information for each subject, including gender, age, T classification, N classification, TNM stage, and overall survival, was obtained retrospectively from clinical records and pathologic reports. TNM status was determined according to the 2002 American Joint Committee on Cancer (AJCC) system. This study was approved by the Medical Ethics and Human Clinical Trial Committee at Chang Gung Memorial Hospital. The subjects included 17 women and 239 men with an average age of 50.9 years (range 26-87 years). Thirty-nine patients were classified as T1, 55 as T2, 64 as T3, and 98 as T4. One hundred and fifty-three patients were classified as N0, 38 as N1, 48 as N2b, 13 as N2c, and 4 as N3. Thirty-four patients were determined to be in stage I, 38 in stage II, 61 in stage III, and 123 in stage IV.

### Immunoblot analysis

For tissue protein extraction, frozen samples were homogenized in RIPA lysis buffer (50 mM Tris-HCl, pH 7.5, 150 mM NaCl, 1% NP-40, 0.5% Na-deoxycholate, and 0.1% SDS). The protein concentration in each sample was estimated by Bio-Rad Protein Assay (Bio-Rad, Hercules, CA, USA). Immunoblotting was performed according to previous reports [27-30]. Antibodies used in this study include Aurora-A (monoclonal; Epitomics, Burlingame, CA, USA), osteopontin (polyclonal; Santa Cruz Biotechnology, Santa Cruz, CA, USA), and β-actin (monoclonal; Santa Cruz Biotechnology, Santa Cruz, CA, USA). The first antibodies were detected by incubation with secondary antibodies conjugated to HRP (Bio/Can Scientific, Mississauga, ON, Canada) and developed using Western Lighting Reagent. The proteins were explored by X-ray films. The protein expression level of Aurora-A in HNSCC tissues was quantified by Bio-Rad Image Lab Software and represented as the densitometric ratio of the targeted protein to β-actin.

### Cell culture, transient transfection, the establishment of stable clones, and luciferase assay

All cell culture-related reagents were purchased from Gibco-BRL (Grand Island, NY, USA). FaDu and SCC4 cells were grown in DMEM containing 10% FBS and 100 U/ml penicillin and streptomycin (Gibco-BRL) HA-vector (pcDNA3.1), and HA-Aurora-A were transiently transfected into cancer cells using Lipofectamine (Invitrogen) according to the manufacturer's instructions. FaDu and SCC4 cells mixed-stably expressing Aurora-A were selected with 400 μg/ml G418 (Calbiochem Novabiochem, San Diego, CA, USA) for two weeks. The cell were then harvested and analyzed for exogenous Aurora-A expressions by Western blotting. 5'-upstream fragments of *Aurora-A* gene (-1~-2000) was amplified from human genomic DNA and verified by sequencing. The PCR fragments were cloned into firefly luciferase reporter vector pGL3-Basic (Promega) NheI and HindIII sites which were designed into the forward and the reverse primers, respectively. For co-transfection experiments, FaDu cells were co-transfected with 100 ng firefly luciferase reporter plasmids (pGL3-Basic or pGL3-Aurora-A), and 10 ng of pRL-TK *Renilla* luciferase internal control plasmid. After 24 h, the luciferase activity was measured using Dual Glo™ Luciferase Assay System (Promega), Two double-stranded synthetic RNA oligomers (5'-GCAGAGAACUGCUACUUAUtt-3'; and GAGUCUACCUAAUUCUGGAtt Ambion; Taipei, Taiwan) deduced from human *Aurora-A*, and one negative control siRNA (#4611G; Ambion) were used in the siRNA experiments.

### Statistical analysis

Several clinicopathological factors were evaluated, including sex, age (less than 59 versus over 60 years), T1, T2 versus T3, T4 stage, N status, and TNM stage. Fisher's exact test was used to evaluate the correlation between the clinicopathological variables and the expression of Aurora-A. A p-value less than 0.05 was considered to be significant in all analyses. The clinicopathological variables and the expression of Aurora-A were taken into account for the analysis of survival based on the Kaplan-Meier method; the statistical significance, defined as a p-value less than 0.05, was assessed by the log-rank test. To determine the effect of particular prognosis factors on survival, a multivariate analysis was performed according to Cox's regression model.

### Supplementary Materials

Materials and methods for Immunohistochemical study, indirect immunofluorescence analysis, RNA extraction, semi-quantitative RT-PCR and quantitative RT-PCR, and Migration and invasion assays are given in Supplementary material.

## SUPPLEMENTARY FIGURES AND MATERIALS


