# Supplementary material for: Aurora-A signaling is activated in advanced stage of squamous cell carcinoma of head and neck cancer and requires osteopontin to stimulate invasive behavior
Source: Oncotarget. 2014 Apr 11;5(8):2243–62. doi: 10.18632/oncotarget.1896 (PMC4039160; doi:10.18632/oncotarget.1896)
Supplement: Supplementary file 1 [file oncotarget-05-2243-s001.pdf]

Supplementary Information

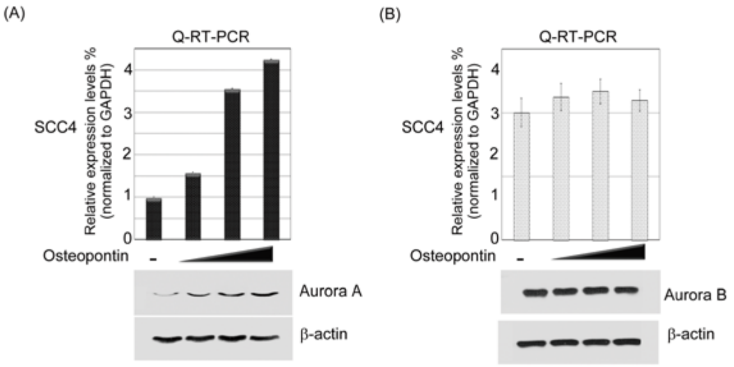

Supplementary Figure 1. The mRNA and protein profiles of Aurora-A, but not Aurora B were enhanced in SCC4 cancer cells upon osteopontin stimulation. (A and B) The mRNA and protein expression levels of Aurora-A and Aurora B were examined by Q-RT-PCR and Western blotting in SCC4 cells in osteopontin dose-dependent manner. The mRNA results were normalized against the expression level of *GAPDH* mRNA in each osteopontin-treated cell. Using the same panel, the total proteins were extracted from SCC4 cells and probed with antibodies against Aurora-A, Aurora B and β-actin. β-actin was used as a control. Data are representative of three independent experiments done in triplicate.

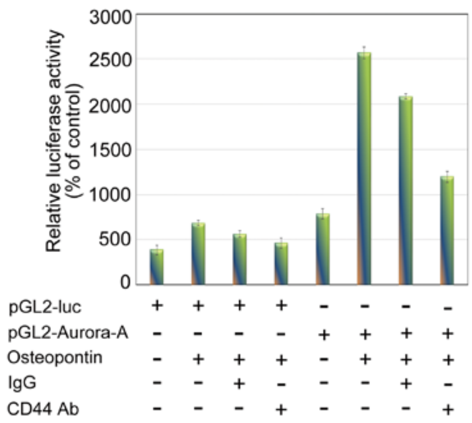

Supplementary Figure 2. The promoter activity of Aurora-A is suupressed by adding CD44 antibody under osteopontin stimulation in human head and neck cancer cell. Luciferase assays were done to detect promoter activity of Aurora-A in transfected FaDu cells in the presence of isotype IgG or CD44 antibody under osteopontin stimulation. The luciferase activity in 1μg of cell lysate was normalized to β-galactosidase activity. Data are representative of three independent experiments done in triplicate.

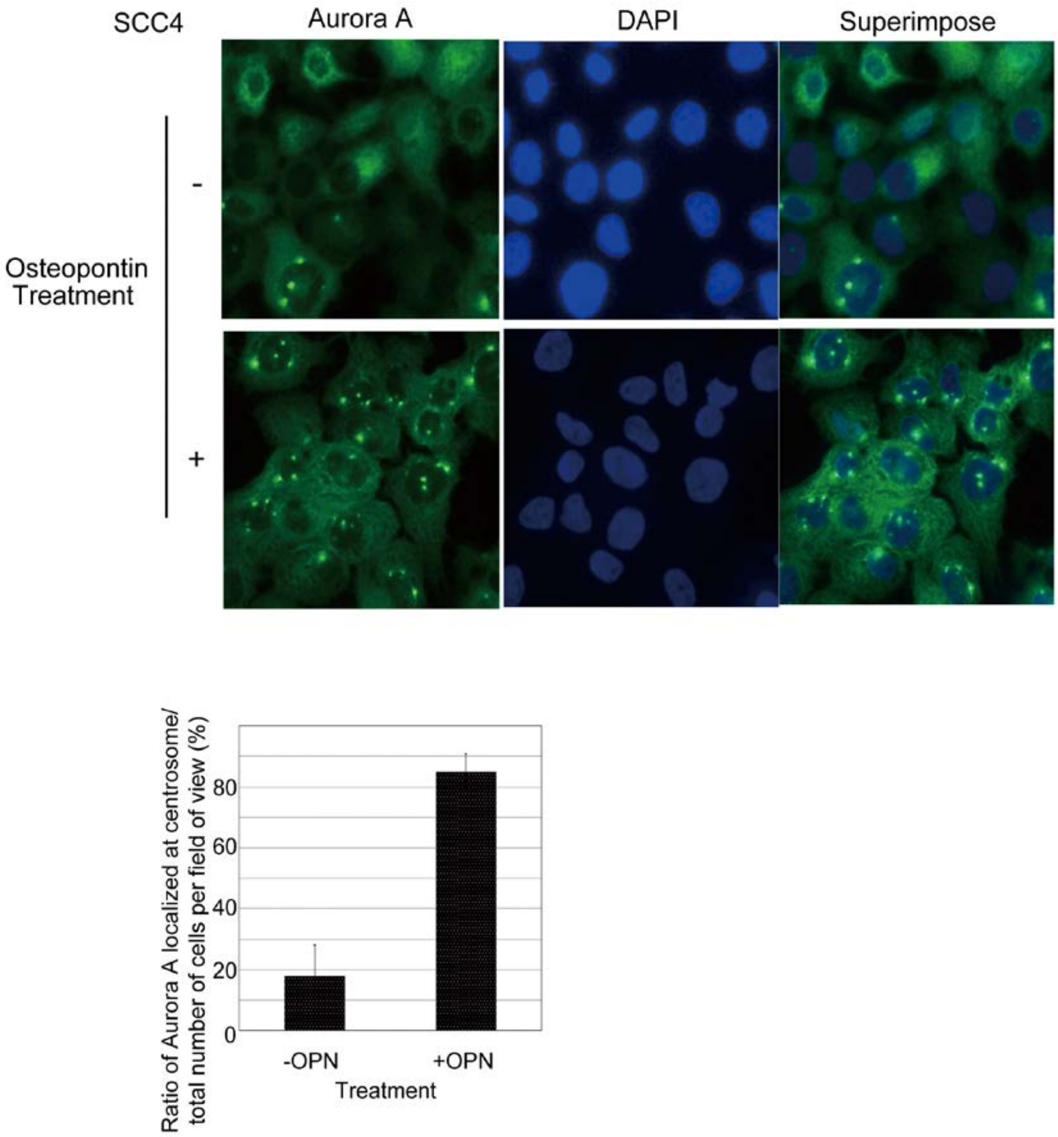

Supplementary Figure 3. Osteopontin induces Aurora-A centrosome localization in SCC4 cells. Immunofluorescence imaging of Aurora-A and nuclei of FaDu cells with or without osteopontin stimulation. Ratio of Aurora-A localized at centrosome was quantified from 20 images per condition.

Supplementary Material

Immunohistochemical study, indirect immunofluorescence analysis and microscopy

Adjacent non-cancerous and tumor HNSCC tissue samples were selected by a pathologist based on diagnosis and microscopic morphology. Tissues were fixed with 10% buffered formalin embedded in paraffin and decalcified in 10% EDTA solution[47, 48]. Representative blocks of the formalin-fixed, paraffin-embedded tissues were cut to 4 mm and deparaffinized with xylene and rehydrated in a series of ethanol washes (100, 90, 80, and 70%). Slides were washed with phosphate-buffered saline (PBS) and treated with 3% H_2O_2 for 30 minutes to block endogenous peroxidase activity. Next, the sections were microwaved in 10 mM citrate buffer, pH 6.0, to unmask the epitopes. After antigen retrieval, the sections were incubated with diluted anti-Aurora-A, anti-osteopontin, and anti-phosphorylated ERK1/2 antibodies for 1 h followed by washing with PBS. Horseradish peroxidase/Fab polymer conjugate (PicTure™-Plus kit; Zymed, South San Francisco, CA, USA) was then applied to the sections for 30 min followed by washing with PBS. Finally, the sections were incubated with diaminobenzidine for 5 min to develop the signals. A negative control was run simultaneously by omitting the primary antibody. The reactivity level of the immunostained tissues was evaluated independently by two pathologists who were blind to the subjects' clinical information. Between 15 and 20 high-power fields were viewed. Criteria were developed for quantitating the immunoreactivities of the Aurora-A staining in both the normal and tumor sections using a score range of 0 to +3, where 0 indicated no positive cell staining, +1 less than 10% positive cell staining, +2 10-30% positive cell staining, and +3 more than 30% positive cell staining. Similarly, the stain intensity was graded as +0, +1, +2, or +3 as previously described. [41] The quantitating the immunoreactivities of the osteopontin and phosphorylated-ERK staining were followed the protocol of Aurora-A. The indirect immunofluorescence staining on the HNSCC biopsies was performed with anti-Aurora-A at

RT for 2 h. The sections were then washed three times with PBST and incubated with goat-anti-mouse-Texas Red and goat-anti-rabbit-FITC (Jackson, ImmunoResearch) at RT for 1 h. After washing with PBST, the sections were mounted with GEL/Mount (biomeda corp, Foster, CA). The fluorescence images on the slips were examined using a confocal microscope (Olympus FV10i).

RNA extraction, semi-quantitative RT-PCR, and quantitative RT-PCR

Tissue samples were frozen in liquid nitrogen and stored at -80°C prior to RNA extraction. The tissues were homogenized using a Mixer Mill Homogenizer (Qiagen, Crawley, West Sussex, UK). Total RNA was prepared from the frozen tissue samples using an RNeasy Mini Kit (Qiagen) according to the manufacturer's instructions. The RNA (2 μg) was then reverse transcribed into cDNA using SuperScript II Reverse Transcriptase (Invitrogen, Carlsbad, CA, USA). PCR was subsequently performed using 1 μL of the reverse transcription products in a total volume of 25 μL with 10-paired HNSCC specimens. The primers used were as follows: *Aurora-A* forward 5'-GCAGATTTTGGGTGGTCAGT-3' and reverse 5'-CAAAAGGAGGCTTCCCAACT-3', and *GAPDH* forward 5'-GAAGGTGAAGGTCGGAGTC-3' and reverse 5'-GAAGATGGTGATGGGATTTC-3'. *GAPDH* was used as an internal control to normalize the relative amount of cDNA in each reaction. The logarithmic phase of amplification for *Aurora-A* was determined during our initial experiments. The reaction mixture contained 10 mM Tris-HCl, pH 8.3, 1.5 mM $MgCl_2$, 50 mM KCl, 200 mM dNTPs, 2 mM each primer, and 1 U of Ex Taq Polymerase (Takara, Shiga, Japan). The PCR program was as follows: 94°C for 1 min, 57°C for 1 min, and 72°C for 2 min for a total of 25 cycles. The RT-PCR products were separated by 2% agarose gel electrophoresis and visualized using 0.5 μg/mL ethidium bromides. The product size for *Aurora-A* was 173 bp. For Q-RT-PCR, *Aurora-A* Taq-Man probe (ABI) were used to perform the study. Data were represented as mean ± s.d. To analyze the distribution of tumor and adjacent non-cancerous parts, we performed the Wilcoxon signed rank test between two

groups for statistical analysis. A *P*-value of less than 0.05 was significant. *GAPDH* (ABI) was used as an internal control for comparison and normalization the data. Assays were performed in triplicate using Applied Biosystems Model 7700 instruments. The mRNA expression level of Aurora-A were quantified by Bio-Rad Image Lab Software and represented as the densitometric ratio of the targeted protein to GAPDH.

Migration, invasion and wound-healing assays

Migration and invasion assays were conducted with FaDu-vehicle, SCC4-vehicle, FaDu/Aurora-A and SCC4/Aurora-A mixed-stable clones using 24-well Transwell chambers (8-µm pore size polycarbonate membrane; Costar, Corning, NY). For the migration (5×10^3) and invasion (1×10^4) assays, cells were suspended in 400 µl of DMEM containing 10% FBS, then seeded into the upper chamber; 600 µl of DMEM containing 10% FBS were added to the outside of the chamber. After being cultured at 37°C under 5% CO_2/95% air for 24 h, the cells on the upper surface of the membrane were removed with a cotton-tipped applicator and the migratory cells on the lower membrane surface were fixed with methanol and stained with Giemsa (Sigma, USA). Cell migration was evaluated by counting the number of FaDu-vehicle, SCC4-vehicle, FaDu/Aurora-A and SCC4/Aurora-A mixed-stable clone cells that had migrated by 200X phase-contrast microscopy on three independent membranes, then normalized against the vehicle cells to determine the relative ratio. For the invasion assays, 80 µg/ml of Matrigel (BD Biosciences) were added to the upper surface of the membrane and allowed to gel at 37°C overnight. A total cells ($1X10^5$) in 400 µl of DMEM containing 10% FBS were seeded into the upper chamber, while 600 µl of DMEM containing 10% FBS were added to the outside of the chamber. The rest of the protocol was the same as that for the migration assays. Vehicle-/HA-Aurora-A-/negative control-/siAurora-A-transfectants were used in the "wound healing" assay to test the alteration of cell migration. Cells were initially seeded uniformly onto 60-mm culture plates with an artificial "wound" carefully created at 0

h. A P-10 pipette tip was used to scratch the sub-confluent cell monolayer. Micro-photographs were taken at 0 and 24 h. Quantitative analysis of the percentage of wound healing was calculated using the distance across the wound at 0 and 24 h, divided by the distance measured at 0 h for each cell line.
